# Supplementary material for: Polymorphisms in Pfkelch13 domains before and after the introduction of artemisinin-based combination therapy in Southwest Nigeria
Source: PLoS One. 2025 Mar 31;20(3):e0316479. doi: 10.1371/journal.pone.0316479 (PMC11957316; doi:10.1371/journal.pone.0316479)
Supplement: Supporting information 2 — (ZIP) [file pone.0316479.s002.zip › 199KN2F_PREMIX_Plate_KELCH2_C09.pdf]

Samples: 12884
Bases: 949
Average spacing: 14

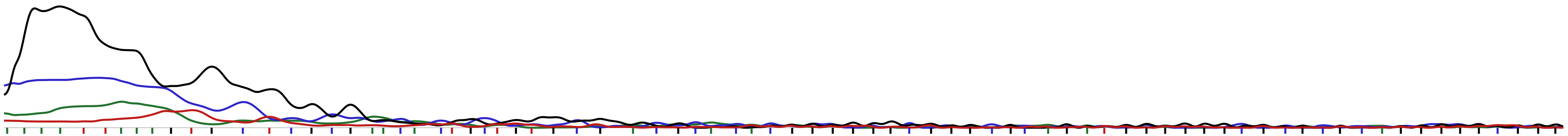

10 20 30 40 50 60 70 80
A A A A T T A A A G T G C T C G C G A A C A C T G C T G T G C G A C G C A C A C G G G G G G C G G G C G C A G A T G G G G G G C G C G C G C A G G G G G C G G G

90 100 110 120 130 140 150 160
C G G G C G T C C C G T G C G G G C G G G T T G A G T C G G C G G G G G G G C G A C G G A G G G G G C T A A C A A A C G G A T T T T T T A C C A A G T A A T G T

170 180 190 200 210 220 230 240
A T C A T G A T C A T T A A C T C A C G T G G C C T A T C T A A A A A T T T G A T C C C T T G T T T A T C T C G G G T G A C A T G T A G A C T A C A A C A T A C T A T T

250 260 270 280 290 300 310 320
A C C A G G A G A A G G A A T C T T T T T G C A G G G T A A A A G T A T G A C T A A T C T G T C A G C A A A T A G C T C C C T A A C A T T C T T A T T A A T A A T G

330 340 350 360 370 380 390 400
G A T G G G T C T C C A T C A G T G C T T C A A C C C A T C T T A T A A G A G A A T T C T C A A G A T G C A T A T A A T C T T G A T A A T G A T G A G C T T T T T

410 420 430 440 450 460 470 480 490
C T A G A T A A A A C A C T A A A T T T T C T A C A G C A A T T A A T G G A A A A G A T A C T T A T C T T T C T A C A A C A A T T C A T C T G C A A T G A T C T T C G

Samples: 12884
Bases: 949
Average spacing: 14

C T T T C G A T T T T G A T T A G A A G G A A A G C G A T T G A T A T A T T A C A T A T C T T A T T T G T T T C T A A A A G A C A G A A A T T C A T T T G A C T C C A G

T A T C A A A A C A G T T T T C T A A A T C T A G A A A C A A C C A G A C T C G T G A A G A G T T C G A A T T C T C A C T G A A C T A G A A A T C C G T T A T C T T T T

T C A C A A C G A A A A T T C C T G A T G T A T G A T G A A A C C A A A T A T T G A G A T G T C G G T C G G T C A C C A G C T A C C A T A T C A T C A A A A T A A G A

A C G C A T G A A A C G A T T G C A T G A T A T A A T T C T C T T T T C A C A A C A T T C G C T G C A G T A C A T G C A G A T G A T G A T G T T T A T A A G T T G G A

T T A A T C A A G A A T C A T C A T T A T A T T A T T G C T G C A T C T A G A T G A T T C T G T T T T T T G G A T G C A G T C G T G T T G A T G G A G T A A T T C

G T A T T A T A A T G A T A A G A G T A G T T A G G T T T A T T A C T G G C C G G A

Samples:	12884
Bases:	949
Average spacing:	14
